# Thymosin β4 promotes hepatoblastoma metastasis via the induction of epithelial-mesenchymal transition

**DOI:** 10.3892/mmr.2015.3359

**Published:** 2015-02-16

**Authors:** XIAOJUN FU, PEIYUAN CUI, FANGFANG CHEN, JIANZHONG XU, LI GONG, LEI JIANG, DAKUN ZHANG, YONGTAO XIAO

**Affiliations:** 1 Departments of Pediatric Surgery, The First Affiliated Hospital of Bengbu Medical College, Bengbu, Anhui 233004, P.R. China; 2 Departments of Hepatobiliary Surgery, The First Affiliated Hospital of Bengbu Medical College, Bengbu, Anhui 233004, P.R. China; 3 Shanghai Institute of Pediatric Research, Shanghai 200092, P.R. China

**Keywords:** hepatoblastoma, thymosin β4, metastasis, epithelial-mesenchymal transition, children

## Abstract

Hepatoblastoma (HB) is the most common malignant hepatic tumor in children and complete surgical resection offers the highest possibility for cure in this disease. Tumor metastasis is the principle obstacle to the development of efficient treatments for patients with HB. The present study aimed to measure the expression levels of thymosin β4 (Tβ4) in liver samples from patients with HB and to investigate the involvement of Tβ4 in HB metastasis. The expression of Tβ4 was significantly higher in liver samples from patients with metastatic HB and in the HepG2 metastatic HB cell line, compared with that in adjacent healthy liver samples and in the L02 healthy hepatic cell line. By contrast, the expression levels of epithelial-cadherin (E-cadherin) and cytosolic accumulation of β-catenin, the two most prominent markers involved in epithelial-mesenchymal transition (EMT), were reduced in liver specimens from patients with metastatic HB compared with that of healthy adjacent control tissue. HepG2 cells were transfected with small interfering-RNA in order to downregulate Tβ4 gene expression. This resulted in a reduced cell migratory capacity compared with control cells. Tβ4 gene expression knockdown significantly inhibited transforming growth factor β1-mediated-EMT *in vitro* by upregulating the expression of E-cadherin. The results of the present study suggested that Tβ4 may promote HB metastasis via the induction of EMT, and that Tβ4 may therefore be a target for the development of novel treatments for patients with HB.

## Introduction

Hepatoblastoma (HB) is a type of liver cancer, which is common in children (1). The primary challenges for the improvement of treatment efficacy include HB metastasis and drug resistance (2,3). Understanding the molecular mechanisms underlying HB metastasis may be useful for the development of novel therapeutic gene targets and the identification of diagnostic biomarkers for HB. Epithelial-mesenchymal transition (EMT) is associated with the progression of a number of epithelial tumors (4–6). EMT consists of a coordinated series of stages, where epithelial cells lose their cell polarity and their cell-cell adhesion, and develop into mesenchymal cells, which results in tumor invasion and metastasis. The process of EMT is essential for HB metastasis and invasion to occur (7,8).

Thymosin β4 (Tβ4), a small peptide originally isolated from calf thymus, is found in human tissues and blood platelets. Tβ4 predominantly functions as a G-actin sequestering factor, modulating the dynamic changes of the actin cytoskeleton. In addition, Tβ4 may be involved in a number of physiological and pathological processes, including the migration of fibroblasts (9,10), endothelial cells and keratinocytes (11,12), and wound healing (13,14). In addition, the prospect of targeting Tβ4 in cancer therapy has recently been proposed. A previous study suggested that Tβ4 was overexpressed in osteosarcoma, colorectal carcinoma and esophageal cancer cells (15). Wang *et al* (16) demonstrated that Tβ4 overexpression suppressed epithelial-cadherin (E-cadherin) expression and led to the increased motility of SW480 human colon carcinoma cells. The present study therefore investigated the involvement of Tβ4 in HB metastasis.

## Materials and methods

### Materials and specimen preparation

Fetal bovine serum (FBS) was purchased from Gibco Life Technologies (Carlsbad, CA, USA). Tβ4 was obtained from Prospec (Rehovot, Israel). Rabbit monoclonal anti-E-cadherin (cat. no 3195), rabbit monocloncal anti-neural-cadherin (anti-N-cadherin; cat. no 13116), rabbit monocloncal anti-β-catenin (cat. no. 9582) and rabbit monocloncal anti-glyceraldehyde 3-phosphate dehydrogenase (anti-GAPDH; cat. no. 9582) antibodies were obtained from Cell Signaling Technology Inc. (Danvers, MA, USA). Rabbit polycloncal anti-Tβ4 antibody (sc-67114, for western blot analysis) was obtained from Santa Cruz Biotechnology, Inc. (Dallas, TX, USA). Rabbit polyclonal anti-Tβ4 antibody (ab14335, for immunohistochemical and immunofluorescence analysis) was purchased from Abcam (Hong Kong, China). Mouse monoclonal anti-α-smooth muscle actin (anti-α-SMA; A5228) was obtained from Sigma-Aldrich (St. Louis, MO, USA). Lipfectamin RNAiMAX was purchased from Invitrogen Life Technologies (Carlsbad, CA, USA). Liver samples from HB and adjacent healthy tissue were obtained from 19 patients with HB at Xinhua Hospital and First Affiliated Hospital of Bengbu Medical College (Anhui, China). This study was performed according to a protocol approved by the faculty of the Medicine Ethics Committee of the First Affiliated Hospital of Bengbu Medical College. Written informed consent (BYKF-D-2009-0914) was obtained from the patients’ guardians prior to specimen collection.

### Immunohistochemistry and immunofluorescence analysis

Immunohistochemistry was performed using a diaminobenzidine (DAB; Dako, Glostrup, Denmark) chromogen method as described previously (17). Specimens were initially incubated using xylol. Endogenous peroxidases were then removed by incubating the samples with 0.3% hydrogen peroxide. The primary antibodies (anti-E-cadherin, 1:400; anti-Tβ4, 1:1,000; and anti-β-catenin, 1:100) were incubated overnight in a chamber with water bottom at 4°C. The slides were washed in phosphate-buffered saline (PBS) and incubated with the horseradish peroxidase (HRP)-conjugated goat anti-rabbit immunoglobulin (Ig)G secondary antibody (1:200; #7074; Cell Signaling Technology, Inc.). Antibody binding was visualized using a liquid DAB Substrate Chromogen System (Dako). In order to conduct an immunofluorescence assay, the cells were fixed with 4% paraformaldehyde. Subsequently, the cells were incubated with the anti-Tβ4 antibody (1:200) and blocked using 3% bovine serum albumin (MP Biomedicals, Auckland, New Zealand). Following three wash stages with PBS, the secondary antibody, conjugated to fluorescein isothiocyanate (1:200; #111-095-045; goat anti-rabbit; Jackson ImmunoResearch, Inc., West Grove, PA, USA), was applied to the cells. The nuclei were counter-stained using 4′, 6-diamidino-2-phenylindole (DAPI; Dojindo, Kumamoto, Japan). The results were visualized using a fluorescence microscope (Eclipse; Nikon, Tokyo, Japan). The nuclei staining and Tβ4 staining were then merged (magnification, ×40).

### Cell culture

The L02 human healthy liver and HepG2 hepatoblastoma cell lines were cultured in Dulbecco’s modified Eagle’s medium (DMEM; Gibco Life Technologies), supplemented with 10% fetal bovine serum (FBS; Gibco Life Technologies) at 37°C in a humidified atmosphere of 5% CO_2_. Tβ4-small interfering RNA (Tβ4-siRNA) duplexes were synthesized by Genepharma Co., Ltd. (Shanghai, China). The siRNA sequences were 5′-CUUCCAAAGAAACGAUUGATT-3′ 5′ - UCGAUAAGUCGAAACUGAATT-3′ and 5′-GAGGUUGGAUCAAGUUUAATT-3′. Tβ4-siRNA transfection was conducted using Lipfectamin RNAiMAX (cat. no. 13778; Life Technologies, Carlsbad, CA, USA) according to the manufacturer’s instructions.

### TGF-β1 EMT in vitro

HepG2 cells were stimulated for 72 h in a conditioned medium containing TGF-β1 (10 ng/ml), supplemented with 0.5% FBS. Total protein was harvested in order to conduct western blot analysis.

### Western blotting

Protein concentrations of cell lysates were determined using a bicinchoninic acid protein assay kit (Pierce, Biotechnology, Inc., Rockford, IL, USA). Protein was separated using electrophoresis on a Novex^®^ 10% Tris-glycine gel (Life Technologies) and transferred onto a nitrocellulose membrane (Life Technologies). The membranes were incubated with the following primary antibodies overnight at 4°C: Anti-E-cadherin (1:1,000), anti-N-cadherin (1:1,000), anti-α-SMA (1:200), anti-β-catenin (1:1,000), anti-Tβ4 (1:200) and anti-GAPDH (1:1,000). The membranes were then incubated with a HRP-conjugated anti-rabbit (1:2,000; cat. no. 7074) and anti-mouse (1:2,000; cat. no. 7076) IgG secondary antibody (Cell Signaling Technology, Inc.) after washing with PBS three times. The antibodies were detected using an enhanced chemiluminescence detection kit (Thermo Fisher Scientific, Waltham, MA, USA) and the Molecular Imager^®^ ChemiDoc™ XRS+ System (Bio-Rad Laboratories, Hercules, CA, USA).

### Wound healing

Confluent cells in 6-well plates were scratched using 100-μl pipette tips. The cells were then incubated at 37°C to allow cell migration into the wound. Following fixation, the number of cells that had migrated into the wound were counted using a microscope (Eclipse Ti; Nikon, Tokyo, Japan). Migration ratio (%) was calculated as the wound width at 24 h/wound width at 0 h.

### Transwell migration assay

Migration of HepG2 cells was determined using 24-well Transwell chambers (Corning Life Sciences, Tewksbury, MA, USA), according to the manufacturer’s instructions. DMEM (500 μl) was added to the lower chambers of the 24-well plate, containing 10% FBS. Cells (1×10^4^/well) were mixed with 100 μl of DMEM without FBS, and the mixture was added to the upper chambers of the 24-well plate. Transwell chambers were incubated at 37°C in a 5% CO_2_ humidified atmosphere for 24 h. Cells that had migrated to the lower surface of the polycarbonate membranes (12-mm pore size) were fixed, stained with crystal violet (Amresco, Solon, OH, USA) and quantified by counting five fields of view using a microscope (Eclipse Ti; Nikon; magnification, ×40).

### Statistical analysis

All data are expressed as the mean ± standard deviation. Statistical significance for comparisons made between two groups was determined using Student’s t-test analysis in SPSS version 13 (SPSS Inc., Chicago, IL, USA).

## Results

### Tβ4 expression is associated with HB metastasis

**Figure 1:**
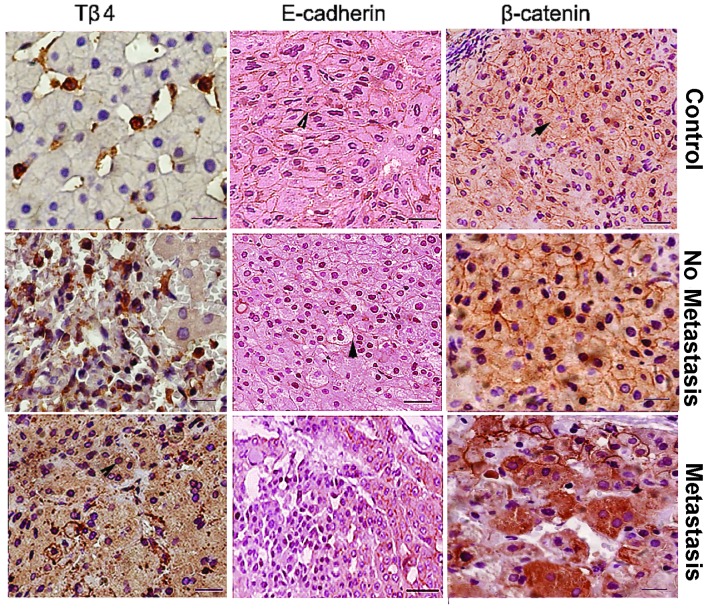
Expression of Tβ4 was associated with HB metastasis. The level of Tβ4 protein expression was higher in patients with HB metastasis compared with that in control and patients with HB without metastasis. HB, hepatoblastoma; Tβ4, thymosin β4.

**Figure 2:**
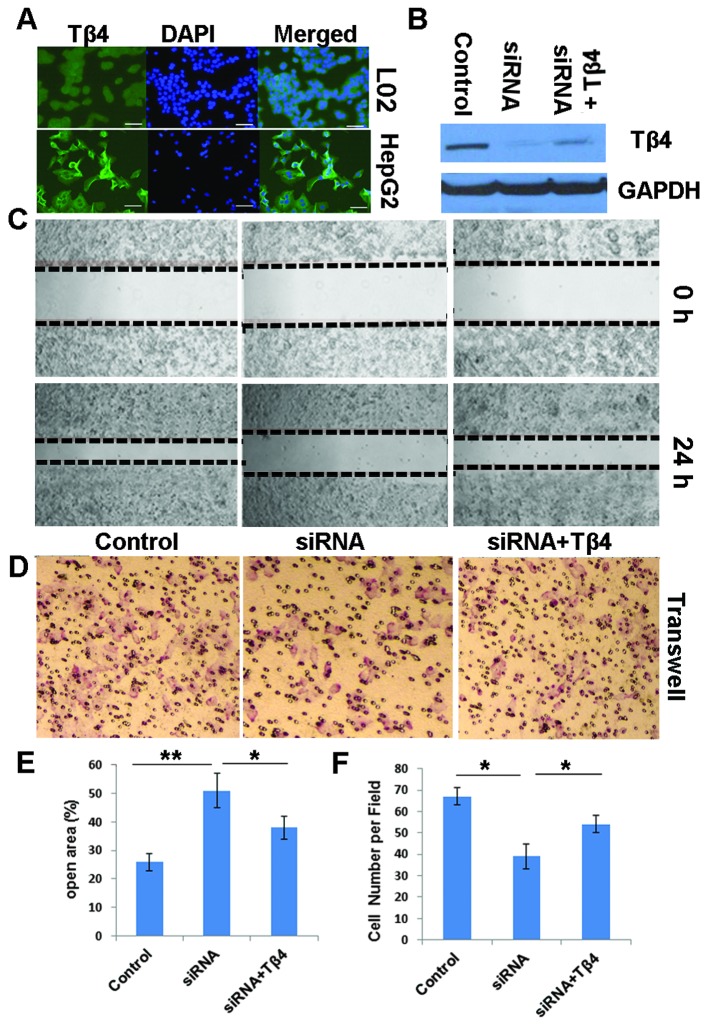
Knockdown of Tβ4 gene expression inhibited the migratory capability of HepG2 cells compared with that of control cells. (A) Immunofluorescence analysis demonstrated that Tβ4 protein was expressed strongly in HepG2 cells compared with that in L02 healthy cells following incubation for 48 h. (B) Tβ4 protein expression decreased following transfection with Tβ4-siRNA. (C and E) Wound healing and (D and F) Transwell migration assays demonstrated that, following Tβ4 gene expression knockdown, HepG2 cell migratory capability was significantly reduced compared with that in control cells. Magnification: ×40. Data are presented as the mean ± standard deviation. ^*^P<0.05 and ^**^P<0.01. Tβ4, thymosin β4; siRNA, small interfering RNA; DAPI, 4′, 6-diamidino-2-phenylindole.

The clinico-pathological characteristics for the 19 patients with HB used in the present study are provided in Tables I and II. The expression of Tβ4 protein in HB tumor tissue samples and adjacent healthy control tissues was analyzed. Tβ4 expression was higher in HB samples compared with the adjacent healthy samples (Fig. 1). No significant associations were observed between Tβ4 expression levels and the gender, age or tumor subtype of patients with HB (Table I). Tβ4 expression was significantly higher in metastatic HB samples compared with the that of the non-metastatic HB samples (91% vs. 25%; P<0.01; Table I). Expression of Tβ4 in metastatic HB specimens was found to be increased, while E-cadherin expression as well as the cytosolic accumulation of β-catenin were reduced in these samples (Fig. 2 and Table II). Overall, these results indicate that upregulation in the expression of Tβ4 may be associated with HB metastasis.

### Expression of Tβ4 promotes HB cell motility

In order to investigate the association between endogenous Tβ4 levels and the migratory capability of HB cells, siRNA was used to target Tβ4 mRNA and knockdown Tβ4 gene expression in HepG2 cells. Silencing of Tβ4 expression in HepG2 cells (Tβ4 siRNA-transfected cells; Fig. 2A and B) resulted in a significant reduction in cell migratory capability compared with that of control HepG2 cells, according to a wound healing assay (P<0.01; Fig. 2C and E). Following treatment with Tβ4 (siRNA + Tβ4 Fig. 2C and E) the migratory capability of HepG2 cells was significantly greater than that of control HepG2 cells, and significantly lower than that of Tβ4 silenced (siRNA) HepG2 cells (P<0.05 and 0.01, respectively). Similar results were observed in the Transwell assays (P<0.05; Fig. 2D and F).

### Tβ4 depletion inhibits EMT in HB cells

**Figure 3:**
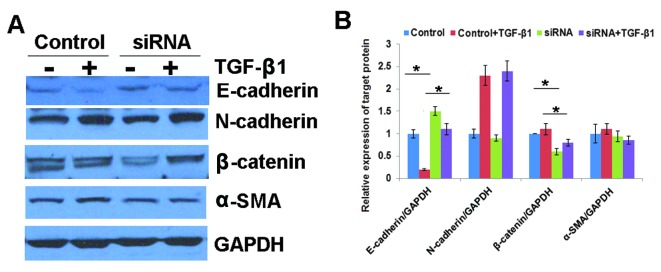
Tβ4 knockdown suppresses the EMT process. (A and B) Downregulated Tβ4 inhibited TGF-β1-induced EMT in HepG2 cells. Tβ4, thymosin β4; siRNA, small interfering RNA; TGF-β1, transforming growth factor β1; E-cadherin, epithelial cadherin; N-cadherin, neural cadherin; GAPDH, glyceralde-hyde 3-phosphate dehydrogenase; SMA, smooth muscle actin.

The results of the present study suggested that downregulated Tβ4 expression may be associated with reduced HepG2 cell migratory capability. Markers of EMT were examined in order to investigate the mechanisms underlying these observations. E-cadherin expression was higher in Tβ4-siRNA-transfected HepG2 cells compared with that in control cells, whereas the expression levels of two mesenchymal markers (β-catenin and α-SMA) were lower in Tβ4-siRNA-transfected HepG2 cells compared with those in control cells (Fig. 3A and B). Since TGF-β1 is involved in the induction of EMT (18), the association between Tβ4 expression and TGF-β1-induced EMT was examined. Following TGF-β1 treatment, the expression of E-cadherin in HepG2 cells was significantly lower and the levels of N-cadherin and α-SMA were significantly higher, compared with those in the control HepG2 cells. Following transfection with Tβ4-siRNA, the expression of genes involved in TGF-β1-induced EMT was significantly reduced in HepG2 cells compared with that in control HepG2 cells (Fig. 3A and B).

## Discussion

Tβ4 is a cellular, actin-sequestering protein, which is associated with angiogenesis induction and the metastatic potential of tumor cells (19-24). The results of the present study suggested that Tβ4 is involved in HB metastasis. Tβ4 expression was significantly higher in HB tissue cells compared with that in healthy adjacent cells (Fig. 1). Statistical analysis demonstrated that Tβ4 expression in tumor tissues was significantly associated with HB-derived lymph node metastasis (Table I and II). Tβ4 gene expression knockdown, using siRNA transfection, resulted in a decrease in the migratory capability of HepG2 cells compared with that in control cells (Fig. 2). Furthermore, the inhibition of Tβ4 expression suppressed the process of TGF-β1-induced EMT in HepG2 cells (Fig. 3).

Tumor metastasis is a multistep process, in which cancer cells disseminate from their primary sites and develop secondary malignant growths at distant sites. The process involves local invasion, intravasation, transportation, extravasation and colonization (25). EMT involves a series of steps, in which cell-cell and cell-extracellular matrix interactions are altered in order to release epithelial cells from the surrounding tissue (26). EMT has been shown to be involved in promoting metastasis in epithelium-derived carcinoma (25). A loss of E-cadherin has been hypothesized to promote β-catenin expression, which binds with the transcription factor, T-cell factor/lymphoid enhancer factor, and modulates gene transcription (27). In the present study, it was found that Tβ4 was upregulated in HB metastatic liver samples; by contrast, E-cadherin expression and the cytosolic accumulation of β-catenin were downregulated in these specimens (Table II and Fig. 1). As the principle binding partner of β-catenin, E-cadherin is involved in the stabilization and promotion of β-catenin expression. E-cadherin and β-catenin are associated with the adhesion and the maintenance of epithelial cell layers. The upregulation of Tβ4 expression may lead to the downregulation of E-cadherin expression, and disrupt actin filaments (28). This may subsequently promote the release of β-catenin from the cell membrane, thereby activating target genes and facilitating HB metastasis. The TGF-β pathway appears to induce EMT (29). In the present study, HepG2 cells were treated with TGF-β1 in order to induce EMT. The expression E-cadherin was lower, and that of N-cadherin and β-catenin was higher, in TGF-β1-treated cells, compared with expression of these molecules in the control cells (Fig. 3). These results suggest that Tβ4 depletion may inhibit EMT in HB cells. Adherent cell locomotion is a highly integrated process, initiated by the forward extension of lamellipodia, followed by repeated cycles of protrusion, adhesion and contraction (30,31). Tβ4 is a candidate regulator of cell protrusion that is involved in protrusion-associated processes, such as actin polymerization and matrix metalloproteinase (MMP) expression. Wang *et al* (16) demonstrated that an increase in the invasiveness of SW480 colon carcinoma cells over-expressing Tβ4, was associated with an increase in MMP-7 expression. The involvement of MMP expression in the processes underlying the association between Tβ4 expression and HB metastasis requires further investigation.

In conclusion, elevated Tβ4 expression in HB cells may promote HB metastasis via the deregulation of EMT.

## Figures and Tables

**Table I:** Characteristics of patients with hepatoblastoma.

Clinical parameters	N	Tβ4 PR (%)	χ^2^	P-value
Gender				
M	14	9 (64)	0.13	>0.05
F	5	3 (60)		
Age				
<3 years	13	8 (61)	0.00	>0.05
≥3 years	6	4 (67)		
Tumor subtype				
Fetal	9	6 (67)		
Embryonal	5	3 (60)	0.23	>0.05
Undifferentiated	2	2 (67)		
Epithelial/mesenchymal	3	1 (50)		
Lymph node metastasis				
Yes	11	10 (91)	8.65	<0.01
No	8	2 (25)		

M, male; F, female; N, number; Tβ4 PR, thymosin β4 positive rate.

**Table II:** Clinicopathological features and results of Tβ4, E-cadherin and β-catenin immunostaining in hepatoblastoma samples.

Gender	Age	Tumor subtype	LNM (Y/N)	Tβ4	EC	Cβ-c
M	8 Ye	Fetal	N	+/−	++	−
M						
M	7 Ye	Fetal	N	+/−	−	−
M			N			
M	5 M	Fetal	N	+/−	+/−	−
M	4 Ye	Embryonal	N	+/−	++	−
M	13 M	Embryonal	N	+/−	+/−	+/−
M	15 M	Undifferentiated	N	+/−	++	−
M	8 M	Undifferentiated	N	++	−	++
F	5 Ye	Epithelial/mesenchymal	N	++	+/−	−
M	11 M	Fetal	Y	++	−	+/−
F	9 M	Fetal	Y	+++	−	−
M	11 M	Fetal	Y	+++	−	+++
M	4 Ye	Fetal	Y	+/−	−	+++
M	6 M	Fetal	Y	+++	−	+/−
F	2 Ye	Fetal	Y	++	−	++
F	4 M	Embryonal	Y	+++	−	++
M	5 Ye	Embryonal	Y	+	−	+++
F	13 M	Embryonal	Y	++	−	++
M	9 M	Epithelial/mesenchymal	Y	+++	−	+/−
M	14 M	Epithelial/mesenchymal	Y	+++	−	++

F, female; M, male; Ye, years; M, months; N, no; Y, yes; Tβ4, thymosin β4; LNM, lymph node metastasis; EC, epithelial-cadherin; Cβ-c, cystolic β-catenin; −, negative; +/−, weak; +, mild; ++, moderate; +++, strong.
